# Integrative Proteo‐Transcriptomic Characterization of Androgenetic Alopecia Identifying ME1‐Mediated PPAR Signaling as a Potential Mediator

**DOI:** 10.1111/jocd.16726

**Published:** 2024-12-13

**Authors:** Ru Dai, Xiaoshuang Yang, Lixin Bao, Xianjie Wu, Zhongfa Lu

**Affiliations:** ^1^ Department of Dermatology Second Affiliated Hospital, Zhejiang University School of Medicine Hangzhou Zhejiang China; ^2^ Department of Dermatology Hailar People's Hospital Hulunbeir Inner Mongolia China

**Keywords:** androgenetic alopecia, mRNA, PPAR, proteomics, transcriptomics

To the Editor,

1

Androgenetic alopecia (AGA) is the most prevalent alopecia worldwide. However, the pathogenesis of AGA remains unclear and previous studies have not utilized multi‐omics approaches to assess AGA [1]. Our study presented the first time integrated transcriptomic and proteomic analysis of bald and haired scalp biopsies from patients with AGA, aiming to identify potential targets and molecular mechanisms.

From June to December 2024, four male patients with AGA were enrolled at the dermatology clinic of Zhejiang University School of Medicine Second Affiliated Hospital. All participants signed an informed consent. The study was approved by the Ethics Committee of Zhejiang University School of Medicine Second Affiliated Hospital (IRB20230380). The participants collection, clinical information of participants, and experimental details are presented in Table S1 and Figure S1 and methods. Through transcriptomic and proteomic analyses, a total of 2565 differentially expressed genes (DEGs) and 315 differentially expressed proteins (DEPs) were identified in bald scalps compared to haired scalps. Gene Ontology (GO) analyses revealed that both DEGs and DEPs were primarily enriched in biological processes such as signal transduction and lipid metabolic process. Kyoto Encyclopedia of Genes and Genomes (KEGG) enrichment of DEGs and Gene Set Enrichment Analysis of DEP showed that DEGs and DEPs were associated with peroxisome, PPAR signaling, and fatty acid metabolism (Figure S2A,B). A further four‐quadrants analysis (Figure S2C) for genes/proteins revealed that those genes/proteins up‐regulated on both the mRNA and the protein levels were enriched in pathways related to metabolism of amino acids and fatty acids. In contrast, genes/proteins consistently down‐regulated were enriched in extracellular matrix. Among genes up‐regulated only on the mRNA level, Golgi activities and structures were detected. By contrast, ribosome activities and structures were enriched among proteins up‐regulated only on the protein level. Furthermore, our integrative KEGG enrichment analyses of differentially expressed mRNA‐protein identified 74 pathways with only PPAR signaling showing significant enrichment in both transcriptomics and proteomics (Figure 1A,B and Table S2). The Venn diagram revealed 32 factors that were significantly regulated at both the gene and protein levels (Figure 1C). Pathway enrichment analysis indicated that the co‐expressed DEGs‐DEPs were also significantly associated with the PPAR signaling pathway through ME1 and PLIN4 (Figure 1D and Table 1), with only ME1 exhibiting similar up‐regulated expression patterns at both gene and protein level.

**FIGURE 1:**
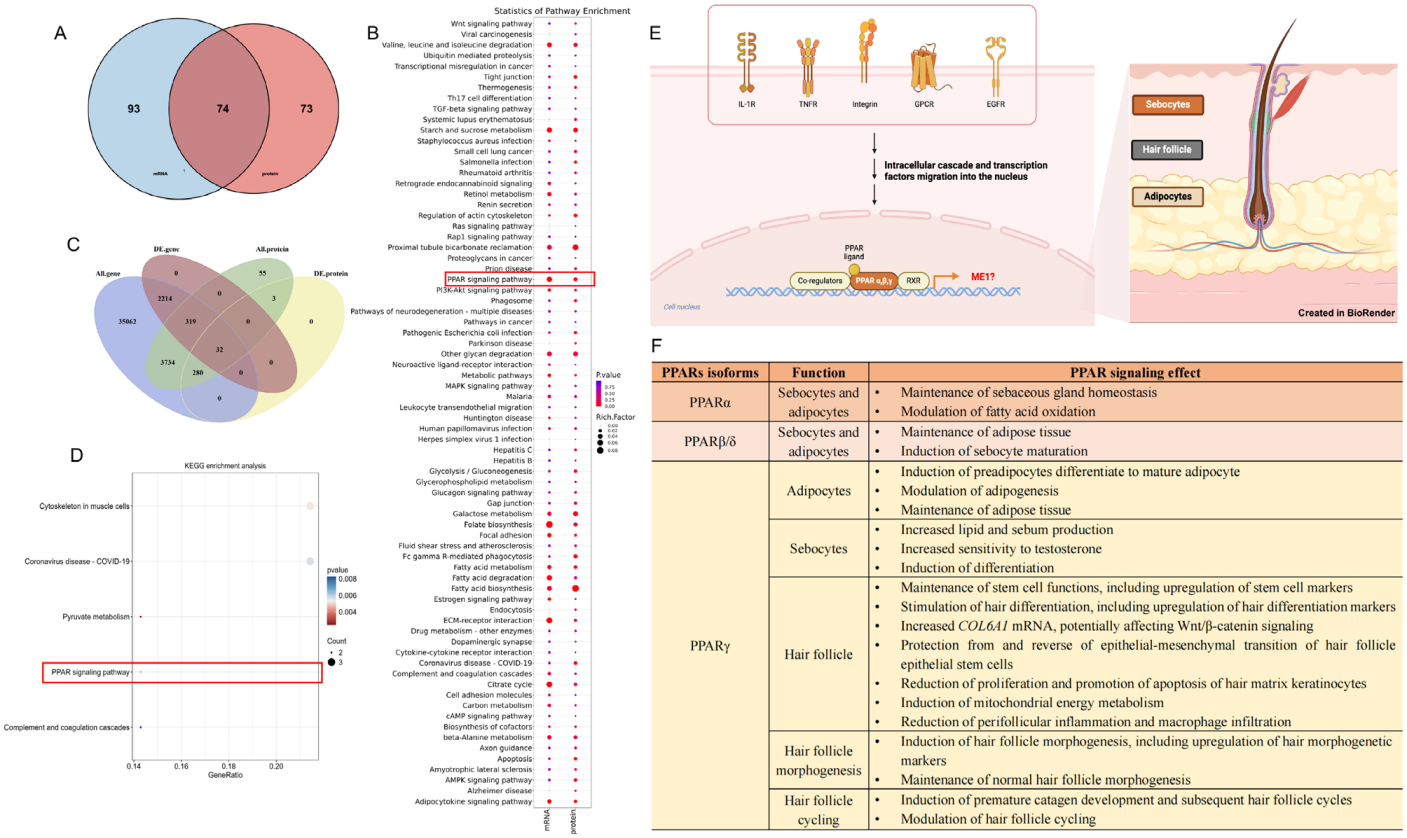
Differential expression analysis of DEGs‐DEPs and enrichment analysis of co‐expressed DEGs‐DEPs indicated significant regulation of the PPAR signaling. (A) Venn diagram of KEGG pathways between DEGs‐DEPs. (B) Bubble plot showing the DEGs and DEPs in enriched KEGG pathways. (C) Joint Venn analysis for co‐expressed DEGs/DEPs. (D) KEGG pathway enrichment analysis of co‐expressed DEGs‐DEPs. (E) An illustration of the PPAR signaling pathway. (F) Biological functions of PPAR signaling in hair follicle, adipose tissue, and sebaceous gland. KEGG, Kyoto Encyclopedia of Genes and Genomes; DEGs, differential expression genes; DEPs, differential expression proteins; PPAR, peroxisome proliferator‐activated receptor.

**TABLE 1:** The list of identified co‐expressed DEPs‐DEGs in PPAR signaling pathway.

Name	Gene ID	UniProt	Description	Pathway definition	Gene expression			Protein expression		
					FC	*p* value	Regulation	FC	*p* value	Regulation
PLIN4	ENSG00000167676	Q96Q06	Perilipin 4	PPAR signaling pathway	0.30	0.026	Down	1.5	0.006	Up
ME1	ENSG00000065833	P48163	Malic enzyme 1	Pyruvate metabolism; metabolic pathways; carbon metabolism: PPAR signaling pathway	2.78	0.001	Up	1.22	0.007	Up

Previous studies have already established a link between PPAR signaling and hair follicle growth and cycling, epithelial stem cell homeostasis, and peri‐follicular inflammation [2, 3, 4, 5]. In Ho et al.'s research, PPAR signaling was implicated in progressive hair miniaturization [6]. However, another study focusing on adipose transcriptome presented a different pattern in a downregulation of PPAR signaling in the scalp of AGA was noted [7]. Altogether, the involvement of the PPAR signaling in AGA may encompass complex activation and inhibition mechanisms. Our research supported the involvement of PPAR signaling in AGA and highlights the important role of ME1. ME1, also known as malic enzyme 1, plays a significant metabolic role in lipogenesis, cholesterol synthesis and cellular redox potential [8]. Previous studies have suggested that ME1 is a PPAR‐related gene/protein involved in energy metabolism [9]. In a mouse model of obesity, ME1 serves as an important physiological regulator of crosstalk among multiple signaling pathways, suggesting its potential role in obesity, diabetes and fatty liver disease in humans. Although previous studies have proved the relationship between lipid metabolism and AGA [10], no studies have explored ME1's role in AGA. The interplay between ME1/PPAR and related pathways in our study is of particular interest because obesity and diabetes are risk factors for AGA. Therefore, it is likely that PPAR signaling may affect hair growth through lipid metabolism with ME1 severing as a key modulator (Figure 1E,F).

Our study was limited by the small sample size, which prevented us to reach a firm conclusion. Additionally, we only assessed mRNA and protein profiles without including metabolic profiles. Incorporating proteomics and metabolomics would allow for the identification of interactions between AGA‐related metabolites and proteins, leading to a deeper understanding of the metabolic regulatory mechanisms in AGA. One key point of our study is the use of paired biopsy scalps from AGA patients as main research subjects, which contain the natural structures of epidermis, dermis, and adipose around the HF. Therefore, the muti‐omics profiles through biopsy are more capable of representing the real biological information especially for the lipid metabolism of AGA in vivo, than samples from plucking or follicular unit extraction.

In conclusion, our study provides a comprehensive characterization of the transcriptomic and proteomic profiles of AGA, emphasizing the pivotal role of PPAR signaling in AGA pathogenesis. Furthermore, we have identified ME1 as a key regulator in the PPAR signaling contributing to AGA development. Overall, our findings provide valuable insight into AGA by suggesting its association with fatty acid or lipid metabolism. However, further research with larger and more diverse cohorts is necessary to confirm these findings. To the best of our knowledge, this is the first study that comprehensively assess molecular changes between bald and haired scalps in AGA at multi‐omics levels.

## Author Contributions

Ru Dai: Data curation, Methodology, Writing – Original draft preparation, Xiaoshuang Yang: Data curation, Methodology, manuscript revision, Linxin Bao: Data curation, manuscript revision, Xianjie Wu: Methodology, manuscript revision, Zhongfa Lu: Methodology, Supervision.

## Ethics Statement

Reviewed and approved by the Ethics Committee of Zhejiang University School of Medicine Second Affiliated Hospital (IRB20230380).

## Consent

The patients in this manuscript have given written informed consent to the publication of their case details.

## Conflicts of Interest

The authors declare no conflicts of interest.

## Supporting information


**Figure S1.** Clinical photos from the affected frontal and unaffected occipital areas of male AGA patients. (A) Patient 1. (B) Patient 2. (C) Patient 3. (D) Patient 4. AGA, androgenetic alopecia.

**Figure S2.** The transcriptomics and proteomics between frontal and occipital scalps from the same patients with AGA. (A) KEGG pathway enrichment analysis of DEGs. The red and blue bars indicate enriched pathways using the up‐regulated and down‐regulated genes in the frontal group, respectively. (B) GSEA enrichment analysis of DEPs. (C) Quadrant analysis of mRNA and proteome data. Scatter plot of (*y*‐axis) the moderated t‐statistics from the differential protein expression analysis of AGA bald scalps vs. non‐bald scalps against (*x*‐axis) the F‐statistics from the differential gene expression analysis of AGA bald scalps vs. non‐bald scalps. Points are colored according to the four quadrants. Enrichment maps show the top 10 enriched Reactome pathways from over‐representation tests of the genes/proteins in each of the four quadrants. In each enrichment map, gene sets with overlapping gene sets are joined by edges. Nodes are colored according to *p*‐value, where gray indicates a higher *p*‐value and pink/orange/green/gray‐blue indicates a lower *p* value. The size of the nodes is proportional to the number of genes in the quadrant within a given gene set. AGA, androgenetic alopecia; KEGG, Kyoto Encyclopedia of Genes and Genome; DEGs, differential expression genes; DEPs, differential expression proteins.

**Table S1.** The characteristics of AGA samples enrolled in this study.
**Table S2**. The list of associated pathways by integrative analyses of differentially expressed mRNA‐protein.

Data S1.

## Data Availability

The data that support the findings of this study are available from the corresponding author upon reasonable request.
