# Supplementary figures and images for: Integrative Proteo‐Transcriptomic Characterization of Androgenetic Alopecia Identifying ME1‐Mediated PPAR Signaling as a Potential Mediator
Source: J Cosmet Dermatol. 2024 Dec 13;24(2):e16726. doi: 10.1111/jocd.16726 (PMC11837229; doi:10.1111/jocd.16726)

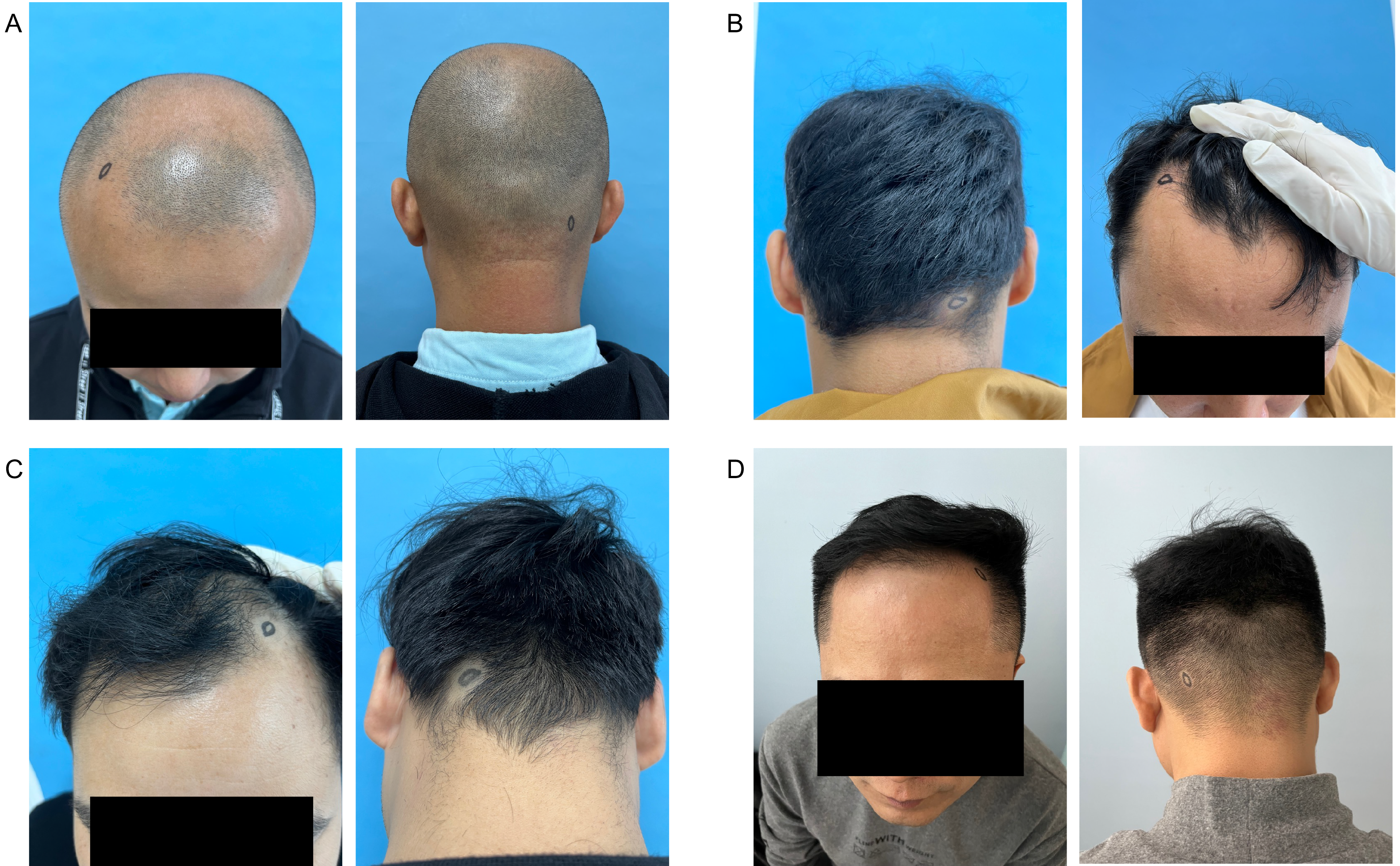

Supplement: Supplementary file 1 — Figure S1. Clinical photos from the affected frontal and unaffected occipital areas of male AGA patients. (A) Patient 1. (B) Patient 2. (C) Patient 3. (D) Patient 4. AGA, androgenetic alopecia. [file JOCD-24-e16726-s001.tif]

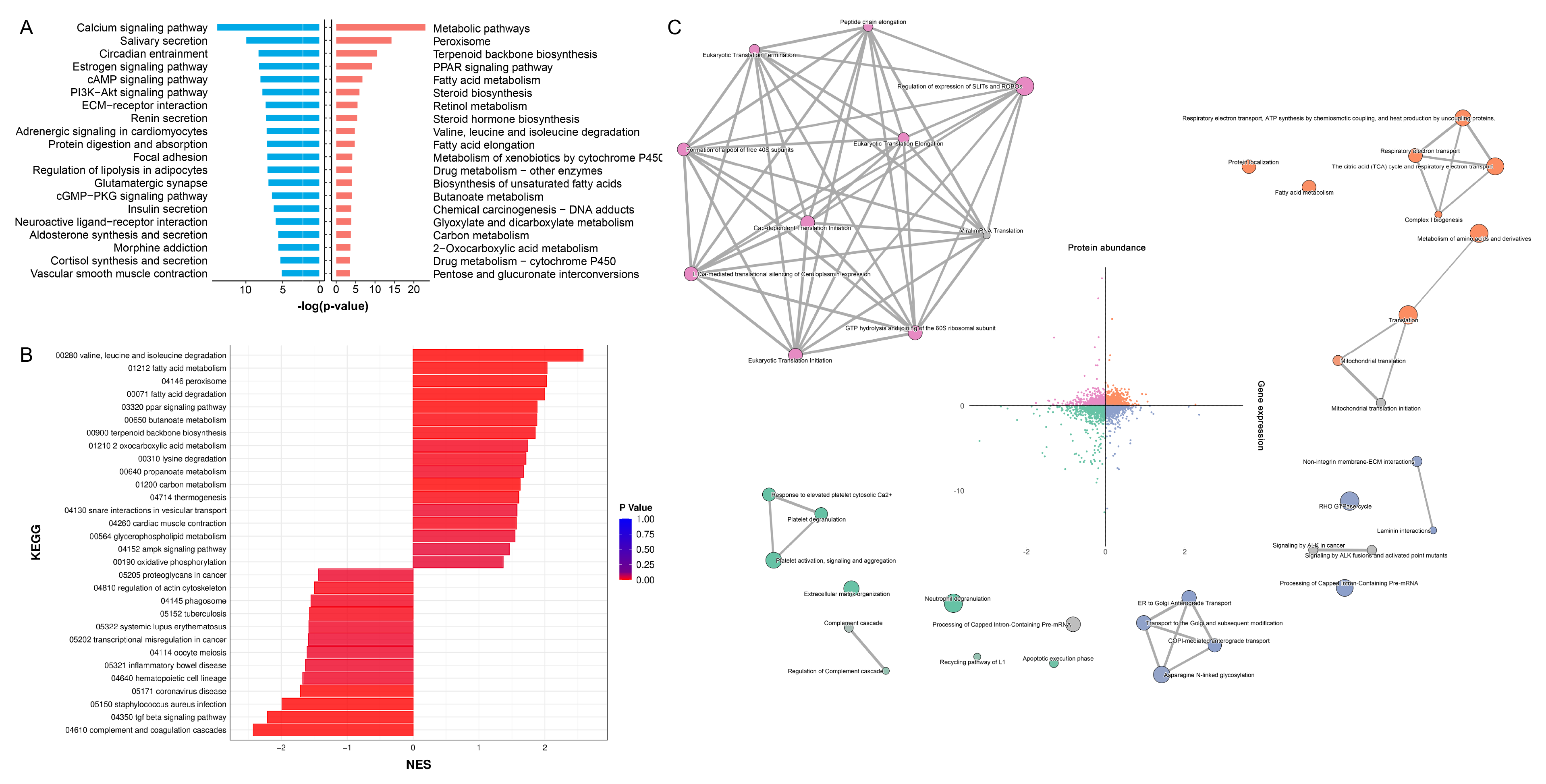

Supplement: Supplementary file 2 — Figure S2. The transcriptomics and proteomics between frontal and occipital scalps from the same patients with AGA. (A) KEGG pathway enrichment analysis of DEGs. The red and blue bars indicate enriched pathways using the up‐regulated and down‐regulated genes in the frontal group, respectively. (B) GSEA enrichment analysis of DEPs. (C) Quadrant analysis of mRNA and proteome data. Scatter plot of (y‐axis) the moderated t‐statistics from the differential protein expression analysis of AGA bald scalps vs. non‐bald scalps against (x‐axis) the F‐statistics from the differential gene expression analysis of AGA bald scalps vs. non‐bald scalps. Points are colored according to the four quadrants. Enrichment maps show the top 10 enriched Reactome pathways from over‐representation tests of the genes/proteins in each of the four quadrants. In each enrichment map, gene sets with overlapping gene sets are joined by edges. Nodes are colored according to p‐value, where gray indicates a higher p‐value and pink/orange/green/gray‐blue indicates a lower p value. The size of the nodes is proportional to the number of genes in the quadrant within a given gene set. AGA, androgenetic alopecia; KEGG, Kyoto Encyclopedia of Genes and Genome; DEGs, differential expression genes; DEPs, differential expression proteins. [file JOCD-24-e16726-s002.tif]
