# Supplementary material for: Integrative Proteo‐Transcriptomic Characterization of Androgenetic Alopecia Identifying ME1‐Mediated PPAR Signaling as a Potential Mediator
Source: J Cosmet Dermatol. 2024 Dec 13;24(2):e16726. doi: 10.1111/jocd.16726 (PMC11837229; doi:10.1111/jocd.16726)
Supplement: Supplementary file 3 — Table S1. The characteristics of AGA samples enrolled in this study. Table S2. The list of associated pathways by integrative analyses of differentially expressed mRNA‐protein. [file JOCD-24-e16726-s004.docx]

Table S1. The characteristic of AGA samples enrolled in this study

| No. | Patient 1 | Patient 2 | Patient 3 | Patient 4 |
| --- | --- | --- | --- | --- |
| Age, years | 34 | 38 | 29 | 46 |
| Duration of AGA, years | 7 | 5 | 5 | 9 |
| Onset of AGA, years | 27 | 33 | 24 | 37 |
| Classification (Norwood-Hamilton Scale) | V | IIIa | IV | IV |
| AGA type | Mixed | Mixed | Mixed | Mixed |
| Comorbidities | None | Type II diabetes | None | None |

Table S2. The list of associated pathways by integrative analyses of differentially expressed mRNA-protein

| **Pathway ID** | **Pathway name** | **KEGG level 1** | **KEGG level 2** | **mRNA KEGG** | | | **Protein KEGG** | | |
| --- | --- | --- | --- | --- | --- | --- | --- | --- | --- |
|  |  |  |  | **Rich factor** | **P value** | **Sig.** | **Rich factor** | **P value** | **Sig.** |
| 4810 | Regulation of actin cytoskeleton | Cellular Processes | Cell motility | 0.01 | 0.27 | ns | 0.02 | 0.00 | ** |
| 5171 | Coronavirus disease - COVID-19 | Human Diseases | Infectious disease: viral | 0.01 | 0.66 | ns | 0.02 | 0.01 | ** |
| 61 | Fatty acid biosynthesis | Metabolism | Lipid metabolism | 0.04 | 0.18 | ns | 0.08 | 0.01 | * |
| 4666 | Fc gamma R-mediated phagocytosis | Organismal Systems | Immune system | 0.01 | 0.56 | ns | 0.03 | 0.02 | * |
| 4964 | Proximal tubule bicarbonate reclamation | Organismal Systems | Excretory system | 0.04 | 0.19 | ns | 0.06 | 0.02 | * |
| 4530 | Tight junction | Cellular Processes | Cellular community - eukaryotes | 0.01 | 0.43 | ns | 0.02 | 0.02 | * |
| 5130 | Pathogenic Escherichia coli infection | Human Diseases | Infectious disease: bacterial | 0.01 | 0.54 | ns | 0.02 | 0.04 | * |
| 52 | Galactose metabolism | Metabolism | Carbohydrate metabolism | 0.03 | 0.24 | ns | 0.04 | 0.04 | * |
| 4210 | Apoptosis | Cellular Processes | Cell growth and death | 0.01 | 0.71 | ns | 0.02 | 0.04 | * |
| **3320** | **PPAR signaling pathway** | **Organismal Systems** | **Endocrine system** | **0.05** | **0.00** | ****** | **0.03** | **0.05** | ***** |
| 500 | Starch and sucrose metabolism | Metabolism | Carbohydrate metabolism | 0.05 | 0.04 | * | 0.04 | 0.05 | ns |
| 5132 | Salmonella infection | Human Diseases | Infectious disease: bacterial | 0.01 | 0.66 | ns | 0.01 | 0.09 | ns |
| 280 | Valine, leucine and isoleucine degradation | Metabolism | Amino acid metabolism | 0.04 | 0.06 | ns | 0.03 | 0.09 | ns |
| 4145 | Phagosome | Cellular Processes | Transport and catabolism | 0.01 | 0.71 | ns | 0.02 | 0.10 | ns |
| 4152 | AMPK signaling pathway | Environmental Information Processing | Signal transduction | 0.01 | 0.65 | ns | 0.02 | 0.10 | ns |
| 1212 | Fatty acid metabolism | Metabolism | Global and overview maps | 0.03 | 0.11 | ns | 0.02 | 0.12 | ns |
| 1100 | Metabolic pathways | Metabolism | Global and overview maps | 0.02 | 0.00 | ** | 0.01 | 0.14 | ns |
| 511 | Other glycan degradation | Metabolism | Glycan biosynthesis and metabolism | 0.04 | 0.20 | ns | 0.04 | 0.15 | ns |
| 4920 | Adipocytokine signaling pathway | Organismal Systems | Endocrine system | 0.03 | 0.03 | * | 0.02 | 0.16 | ns |
| 4151 | PI3K-Akt signaling pathway | Environmental Information Processing | Signal transduction | 0.02 | 0.08 | ns | 0.01 | 0.16 | ns |
| 10 | Glycolysis / Gluconeogenesis | Metabolism | Carbohydrate metabolism | 0.01 | 0.44 | ns | 0.02 | 0.16 | ns |
| 5020 | Prion disease | Human Diseases | Neurodegenerative disease | 0.01 | 0.75 | ns | 0.01 | 0.17 | ns |
| 4510 | Focal adhesion | Cellular Processes | Cellular community - eukaryotes | 0.03 | 0.01 | ** | 0.01 | 0.17 | ns |
| 4714 | Thermogenesis | Organismal Systems | Environmental adaptation | 0.01 | 0.61 | ns | 0.01 | 0.20 | ns |
| 790 | Folate biosynthesis | Metabolism | Metabolism of cofactors and vitamins | 0.08 | 0.02 | * | 0.03 | 0.21 | ns |
| 5160 | Hepatitis C | Human Diseases | Infectious disease: viral | 0.01 | 0.76 | ns | 0.01 | 0.22 | ns |
| 5322 | Systemic lupus erythematosus | Human Diseases | Immune disease | 0.00 | 0.85 | ns | 0.01 | 0.22 | ns |
| 4540 | Gap junction | Cellular Processes | Cellular community - eukaryotes | 0.01 | 0.55 | ns | 0.02 | 0.22 | ns |
| 4512 | ECM-receptor interaction | Environmental Information Processing | Signaling molecules and interaction | 0.06 | 0.00 | ** | 0.02 | 0.23 | ns |
| 5222 | Small cell lung cancer | Human Diseases | Cancer: specific types | 0.01 | 0.56 | ns | 0.02 | 0.23 | ns |
| 5165 | Human papillomavirus infection | Human Diseases | Infectious disease: viral | 0.01 | 0.23 | ns | 0.01 | 0.24 | ns |
| 4360 | Axon guidance | Organismal Systems | Development and regeneration | 0.01 | 0.78 | ns | 0.01 | 0.26 | ns |
| 410 | beta-Alanine metabolism | Metabolism | Metabolism of other amino acids | 0.03 | 0.22 | ns | 0.02 | 0.27 | ns |
| 20 | Citrate cycle (TCA cycle) | Metabolism | Carbohydrate metabolism | 0.06 | 0.03 | * | 0.02 | 0.28 | ns |
| 5014 | Amyotrophic lateral sclerosis | Human Diseases | Neurodegenerative disease | 0.01 | 0.65 | ns | 0.01 | 0.29 | ns |
| 4922 | Glucagon signaling pathway | Organismal Systems | Endocrine system | 0.01 | 0.60 | ns | 0.01 | 0.30 | ns |
| 5323 | Rheumatoid arthritis | Human Diseases | Immune disease | 0.01 | 0.79 | ns | 0.01 | 0.33 | ns |
| 71 | Fatty acid degradation | Metabolism | Lipid metabolism | 0.05 | 0.05 | * | 0.01 | 0.38 | ns |
| 5144 | Malaria | Human Diseases | Infectious disease: parasitic | 0.02 | 0.36 | ns | 0.01 | 0.41 | ns |
| 5418 | Fluid shear stress and atherosclerosis | Human Diseases | Cardiovascular disease | 0.01 | 0.70 | ns | 0.01 | 0.42 | ns |
| 4144 | Endocytosis | Cellular Processes | Transport and catabolism | 0.00 | 0.92 | ns | 0.01 | 0.42 | ns |
| 1240 | Biosynthesis of cofactors | Metabolism | Global and overview maps | 0.01 | 0.40 | ns | 0.01 | 0.43 | ns |
| 830 | Retinol metabolism | Metabolism | Metabolism of cofactors and vitamins | 0.03 | 0.12 | ns | 0.01 | 0.48 | ns |
| 4310 | Wnt signaling pathway | Environmental Information Processing | Signal transduction | 0.01 | 0.76 | ns | 0.01 | 0.50 | ns |
| 4924 | Renin secretion | Organismal Systems | Endocrine system | 0.01 | 0.44 | ns | 0.01 | 0.52 | ns |
| 4610 | Complement and coagulation cascades | Organismal Systems | Immune system | 0.02 | 0.22 | ns | 0.01 | 0.54 | ns |
| 5012 | Parkinson disease | Human Diseases | Neurodegenerative disease | 0.00 | 0.90 | ns | 0.01 | 0.54 | ns |
| 5022 | Pathways of neurodegeneration - multiple diseases | Human Diseases | Neurodegenerative disease | 0.01 | 0.64 | ns | 0.01 | 0.55 | ns |
| 5010 | Alzheimer disease | Human Diseases | Neurodegenerative disease | 0.00 | 0.97 | ns | 0.01 | 0.55 | ns |
| 983 | Drug metabolism - other enzymes | Metabolism | Xenobiotics biodegradation and metabolism | 0.01 | 0.50 | ns | 0.01 | 0.56 | ns |
| 564 | Glycerophospholipid metabolism | Metabolism | Lipid metabolism | 0.01 | 0.62 | ns | 0.01 | 0.57 | ns |
| 4010 | MAPK signaling pathway | Environmental Information Processing | Signal transduction | 0.01 | 0.27 | ns | 0.01 | 0.58 | ns |
| 5016 | Huntington disease | Human Diseases | Neurodegenerative disease | 0.01 | 0.14 | ns | 0.01 | 0.59 | ns |
| 5203 | Viral carcinogenesis | Human Diseases | Cancer: overview | 0.00 | 0.89 | ns | 0.01 | 0.62 | ns |
| 4350 | TGF-beta signaling pathway | Environmental Information Processing | Signal transduction | 0.01 | 0.57 | ns | 0.01 | 0.67 | ns |
| 5150 | Staphylococcus aureus infection | Human Diseases | Infectious disease: bacterial | 0.01 | 0.27 | ns | 0.01 | 0.69 | ns |
| 4120 | Ubiquitin mediated proteolysis | Genetic Information Processing | Folding, sorting and degradation | 0.01 | 0.69 | ns | 0.01 | 0.70 | ns |
| 4670 | Leukocyte transendothelial migration | Organismal Systems | Immune system | 0.01 | 0.62 | ns | 0.01 | 0.70 | ns |
| 1200 | Carbon metabolism | Metabolism | Global and overview maps | 0.02 | 0.26 | ns | 0.01 | 0.71 | ns |
| 4659 | Th17 cell differentiation | Organismal Systems | Immune system | 0.01 | 0.78 | ns | 0.01 | 0.76 | ns |
| 4728 | Dopaminergic synapse | Organismal Systems | Nervous system | 0.01 | 0.69 | ns | 0.00 | 0.77 | ns |
| 4723 | Retrograde endocannabinoid signaling | Organismal Systems | Nervous system | 0.02 | 0.15 | ns | 0.00 | 0.77 | ns |
| 4915 | Estrogen signaling pathway | Organismal Systems | Endocrine system | 0.02 | 0.05 | ns | 0.00 | 0.78 | ns |
| 5161 | Hepatitis B | Human Diseases | Infectious disease: viral | 0.01 | 0.76 | ns | 0.00 | 0.82 | ns |
| 5202 | Transcriptional misregulation in cancer | Human Diseases | Cancer: overview | 0.01 | 0.50 | ns | 0.00 | 0.83 | ns |
| 4514 | Cell adhesion molecules | Environmental Information Processing | Signaling molecules and interaction | 0.01 | 0.36 | ns | 0.00 | 0.85 | ns |
| 5205 | Proteoglycans in cancer | Human Diseases | Cancer: overview | 0.01 | 0.26 | ns | 0.00 | 0.88 | ns |
| 4015 | Rap1 signaling pathway | Environmental Information Processing | Signal transduction | 0.01 | 0.52 | ns | 0.00 | 0.89 | ns |
| 4014 | Ras signaling pathway | Environmental Information Processing | Signal transduction | 0.00 | 0.86 | ns | 0.00 | 0.91 | ns |
| 5200 | Pathways in cancer | Human Diseases | Cancer: overview | 0.01 | 0.47 | ns | 0.00 | 0.92 | ns |
| 4024 | cAMP signaling pathway | Environmental Information Processing | Signal transduction | 0.01 | 0.27 | ns | 0.00 | 0.92 | ns |
| 4060 | Cytokine-cytokine receptor interaction | Environmental Information Processing | Signaling molecules and interaction | 0.01 | 0.50 | ns | 0.00 | 0.95 | ns |
| 4080 | Neuroactive ligand-receptor interaction | Environmental Information Processing | Signaling molecules and interaction | 0.01 | 0.57 | ns | 0.00 | 0.97 | ns |
| 5168 | Herpes simplex virus 1 infection | Human Diseases | Infectious disease: viral | 0.00 | 0.98 | ns | 0.00 | 0.99 | ns |

*P < 0.05, **P < 0.01
