# Supplementary material for: Integrative Proteo‐Transcriptomic Characterization of Androgenetic Alopecia Identifying ME1‐Mediated PPAR Signaling as a Potential Mediator
Source: J Cosmet Dermatol. 2024 Dec 13;24(2):e16726. doi: 10.1111/jocd.16726 (PMC11837229; doi:10.1111/jocd.16726)
Supplement: Supplementary file 4 — Data S1. [file JOCD-24-e16726-s003.zip › revised supplementary methods.docx]

**Supplementary materials**

**1 Patients and sample collection**

The study included four male subjects aged 29 to 46, diagnosed with Hamilton-Norwood Class III to V AGA. They were recruited in November 2023, and the diagnosis was verified by a dermatologist. Patient recruitment was voluntary. Those who met the diagnostic criteria for AGA (Hamilton-Norwood Class III to VI) and were willing to participate were selected. All participants signed an informed consent and were notified about the study before participation. They agreed to have scalp biopsies taken from both bald and non-bald regions. Participants received compensation as an incentive. Additionally, all participants refrained from any systemic or topical treatments for at least three months prior to the biopsy.

The study was approved by the Ethics Committee of Zhejiang University School of Medicine Second Affiliated Hospital (IRB20230380), and all the procedures were performed according to the principles set out in the World Medical Association Declaration of Helsinki and the Department of Health and Human Services Belmont Report. The scalp tissues were obtained from the frontal and occipital sites of above patients undergoing excisional biopsy. A scalp tissue sample sized about 4-6 mm, including both the hair follicle and the subcutaneous tissue, was harvested from each bald and non-bald site. The whole skin tissues were stored in a -80℃ freezer immediately for subsequent experiments.

**2 RNA extraction, RNA-sequencing and bioinformatics analyses**

**2.1 RNA extraction**

Total RNA from scalp tissues were extracted using Trizol (Thermo Fisher) following the manufacturer’s instructions. Extracted RNA was quantified using NanoDrop ND-1000 (NanoDrop, Wilmington, DE), and RNA integrity was assessed by Agilent Bioanalyzer 2100 with RIN number >7.0, and confirmed by electrophoresis with denaturing agarose gel.

**2.2 Library preparation**

All four bald biopsies and four non-bald biopsies from four AGA patients were subjected to RNA-sequencing. RNA-seq library preparation was performed with 2μg of RNA per sample. Sequencing libraries of mRNA were performed on the illumina Novaseq 6000 platform by LC Bio Technology CO., Ltd (Hangzhou, China). Sequencing was performed on Illumina Hiseq 2500 platform by LC Bio Technology CO., Ltd (Hangzhou, China).

**2.3 Bioinformatics analysis of mRNAs**

Fastp software (https://github.com/OpenGene/fastp) were used to remove the reads that contained adaptor contamination, low quality bases and undetermined bases with default parameter. Then sequence quality was also verified using Fastp. We used HISAT2 (https://ccb.jhu.edu/software/hisat2) to map reads to the human reference. The mapped reads of each sample were assembled using StringTie (https://ccb.jhu.edu/software/stringtie) with default parameters. Then, all transcriptomes from all samples were merged to reconstruct a comprehensive transcriptome using gffcompare (https://github.com/gpertea/gffcompare/). After the final transcriptome was generated, StringTie and was used to estimate the expression levels of all transcripts. StringTie was used to perform expression level for mRNAs by calculating FPKM (FPKM = [total_exon_fragments / mapped_reads(millions) × exon_length(kB)]). The differentially expressed genes (DEGs) were selected with fold change > 2 or fold change < 0.5 and with parametric F-test comparing nested linear models (*P* value < 0.05) by R package edgeR (<https://bioconductor.org/packages/release/bioc/html/edgeR.html>). Enrichment analysis was conducted using “clusterprofiler” R package including Gene Ontology (GO) and Kyoto Encyclopedia of Genes and Genomes (KEGG), in which GO contained three items: biological process (BP), cellular component (CC) and molecular function (MF). KEGG enrichment analysis provided differential expression genes that were statistical enrichment in KEGG pathways.

**3 Protein extraction, TMT-labeled proteomics, LC-MS/MS Analysis and bioinformatics analyses**

**3.1 Protein extraction, TMT labeling and quantification**

Protein extraction, TMT labeling and quantification of all samples were performed by LC Bio Technology CO., Ltd (Hangzhou, China). Briefly, 100μg peptide mixture of each sample was labeled using TMT reagent according to the manufacturer’s instructions (Thermo Fisher Scientific), and fractionated by reversed phase (RP) chromatography using the Agilent 1260 infinity II HPLC.

**3.2 LC-MS/MS Analysis**

Proteomics sequencing services were provided by LC Bio Technology CO., Ltd (Hangzhou, China). Each fraction was injected for nanoLC-MS/MS analysis on a Q Exactive plus mass spectrometer (Thermo Fisher Scientific). Then the MS/MS raw files were processed using MASCOT engine (Matrix Science, London, UK; version 2.6) embedded into Proteome Discoverer 2.2, and searched against the Uniprot database. Proteins with Fold change>1.2 and *P* value <0.05 (Student’s t test) were considered to be differentially expressed proteins (DEPs).

**3.3 Bioinformatics analysis of protein sequences**

Venn diagrams, volcano plots, and hierarchical clustering were used to characterize the DEPs. The subcellular localization of proteins was predicted using WoLF PSORT. The protein domain was annotated using Inter-ProScan software from the Pfam, ProDom and SMART databases. Furthermore, the functional categorizations of DEPs were performed by GO term using Blast2GO, and their enrichment metabolic pathways were annotated by KEGG pathways and Gene Set Enrichment Analysis (GSEA).

**4 Integrative bioinformatics analyses of transcriptomics and proteomics**

**4.1 mRNA-protein correction analysis**

Matched genes and their corresponding protein from scalps were correlated. The correlation was performed using the log2-fold changes of protein levels between bald samples and the median of non-bald samples for mRNA and protein data. Uniprot accessions were mapped to Symbol gene IDs. For the mRNA-protein correlation, 4365 genes were included. mRNA-protein correlations were assessed using Spearman correlation tests.

**4.2 Differential expression analysis of transcriptomics and proteomics**

For the assessment of dysregulated genes and proteins in AGA, we performed differential expression analysis as described above between bald and non-bald scalps for transcriptomics and proteome data. To compare the dysregulated genes/proteins between transcriptome data and proteome data, Uniprot accessions were mapped to Symbol gene IDs, resulting in 4365 comparable genes/proteins. Dysregulated pathways were identified using a quadrant analysis, by performing over-representation tests. For proteome data, the corresponding sets of detected proteins were used as background for over-representation tests. *P* ≤ 0.05 was considered statistically significant. Pathway analysis results were represented as barplots, dotplots or enrichment maps. A further KEGG enrichment analysis of DEGs/DEPs was performed to identify the target pathway.

**4.3 Enrichment analysis of co-expressed DEGs-DEPs**

The co-expressed DEGs/DEPs were detected by the Venn analysis. Next, the functional enrichment analyses of co-expressed DEPs-DEGs were performed by GO term and KEGG database.
